# Functional extreme learning machine

**DOI:** 10.3389/fncom.2023.1209372

**Published:** 2023-07-11

**Authors:** Xianli Liu, Guo Zhou, Yongquan Zhou, Qifang Luo

**Affiliations:** ^1^College of Artificial Intelligence, Guangxi University for Nationalities, Nanning, China; ^2^Department of Science and Technology Teaching, China University of Political Science and Law, Beijing, China; ^3^Xiangsihu College of Gunagxi University for Nationalities, Nanning, Guangxi, China; ^4^Guangxi Key Laboratories of Hybrid Computation and IC Design Analysis, Nanning, China

**Keywords:** FN, ELM, functional equation, parameter learning algorithm, FELM

## Abstract

**Introduction:**

Extreme learning machine (ELM) is a training algorithm for single hidden layer feedforward neural network (SLFN), which converges much faster than traditional methods and yields promising performance. However, the ELM also has some shortcomings, such as structure selection, overfitting and low generalization performance.

**Methods:**

This article a new functional neuron (FN) model is proposed, we takes functional neurons as the basic unit, and uses functional equation solving theory to guide the modeling process of FELM, a new functional extreme learning machine (FELM) model theory is proposed.

**Results:**

The FELM implements learning by adjusting the coefficients of the basis function in neurons. At the same time, a simple, iterative-free and high-precision fast parameter learning algorithm is proposed.

**Discussion:**

The standard data sets UCI and StatLib are selected for regression problems, and compared with the ELM, support vector machine (SVM) and other algorithms, the experimental results show that the FELM achieves better performance.

## 1. Introduction

Artificial neural network (ANN) is a parallel computing system which simulates human brain activity by using widely interconnected neuron structure and certain learning rules. Because of its strong self-learning, associative memory, adaptive and fault-tolerant ability, it can easily detect the complex nonlinear relationship between the dependent variable and the independent variables, and has large-scale parallel computing ability. Therefore, it has become a popular and useful model for classification, clustering, pattern recognition and prediction in many disciplines, and is a powerful tool to solve problems that cannot be solved by many traditional methods (Abiodun et al., 2018).

Artificial neural network (ANN) has gone through four stages of development, and hundreds of models have been established so far. It has achieved great success in applied research fields such as handwriting recognition (Baldominos et al., 2018), image annotation (Afridi et al., 2018) and speech recognition (Gautam and Sharma, 2019), et al. However, most ANNs are only simple simulation of biological networks, so they often appear inadequate in dealing with big data and complex tasks, and cannot be satisfactory in both processing speed and calculation accuracy. Among the hundreds of neural network models, traditional training algorithms are usually gradient-based, such as back-propagation neural network (BP) (Werbos, 1974). BP algorithm has been widely used in many fields because of its easy understanding and implementation. However, that gradient-based algorithm is easy to converge to the local minimum and cannot obtain the global optimal solution, because the solution it obtains is sensitive to the initial parameters and depends on the complexity of the feature space, and the iterative learning of the BP algorithm makes the convergence speed too slow. In recent years, Huang et al. proposed for the first time a single hidden layer feedforward neural network learning algorithm called Extreme Learning Machine (ELM) (Huang et al., 2006), which breaks through the commonly used feedforward neural network learning theories and methods. Compared with support vector machine (SVM) (Cortes and Vapnik, 1995), ELM tends to achieve higher classification accuracy with lower computational complexity (Li et al., 2019). Since ELM has the advantages of high learning accuracy, easy to use, easy to implement, and fast learning speed, it has been applied widely in ELM self-encoder (Yimin Yang and Jonathan, 2018), handwriting recognition (Tang et al., 2016), regression and classification (Huang et al., 2012), big data analysis (Sun et al., 2017), and many improved algorithms of ELM (Zong et al., 2013; Geng et al., 2017; Sattar et al., 2019; Gong et al., 2021; Kardani et al., 2021) have also emerged to deal with specific problems. Studies have shown that ELM, especially in some applications, has the advantages of simple structure, short training times, and high calculation accuracy compared with popular deep learning, and the obtained solution is the only optimal solution, which ensures the generalization performance of the network.

The extreme learning machine (ELM) theory has attracted extensive attention by scholars all over the world since it was proposed (Huang et al., 2012; Tang et al., 2016; Sun et al., 2017; Yimin Yang and Jonathan, 2018), and a lot of achievements have been made in its theoretical and applied research. Kärkkäinen (2019) proposed an ELM which conducts ridge regression using a distance-based, the experimental results show that the over-learning with the distance-based basis is avoided in the classification problem. Atiquzzaman and Kandasamy (2018) successfully used ELM for hydrological flow series prediction. Golestaneh et al. (2018) presented a fuzzy wavelet ELM, and its performance is better than ELM. Yaseen et al. (2019) used the enhanced extreme learning machine for river flow forecasting. Pacheco et al. (2018) used restricted Boltzmann machine to determine the input weights of ELM, which greatly optimized the performance of ELM. Christou et al. (2018) proposed a hybrid ELM method for neural networks, which is applied to a series of regression and classification problems. Murli et al. (2018) applied extreme learning machine to microgrid protection under wind speed intermittency. Artem and Stefan (2017) applied ELM to the credit evaluation of user credit cards, indicating that it is a valuable alternative to other credit risk modeling methods. Henríquez and Ruz (2019) used ELM to reduce the noise of near-infrared spectroscopy data, which was successfully applied. Mohammed et al. (2018) proposed an improved ELM based on competitive group optimization and applied it to medical diagnosis. Lima et al. (2017) proposed a variable complexity online sequential ELM, which was successfully used for streamflow prediction. Paolo and Roberto (2017) applied ELM to inverse reactor kinetics, and the experimental results show that ELM application has great potential. Ozgur and Meysam (2018) compared the performance of wavelet ELM and wavelet neural networks. Vikas and Balaji (2020) proposed a PIELM and successfully applied it to solve partial differential equations; Peter and Israel (2020) proposed a new morphological/linear perceptron ELM and implemented fast classification problems.

So far, the ELM has been widely used in industry, agriculture, military, medicine and other fields. Although the research on ELM has made a lot of achievements, from the classification of achievements, there are many application achievements and few theoretical achievements, which greatly limit the application scope of ELM. In particular, there are still the following shortcomings in the ELM theory:

(1) The weights randomly determined by hidden layer neurons have a great impact on the classification performance of the network, and the number of hidden layer neurons cannot be calculated by an effective algorithm. Although some researchers have proposed some optimization algorithms about ELM, these algorithms transform the steps to determine the number of hidden layer neurons into optimization problems, which are cumbersome and time-consuming.
(2) In the learning and training of ELM, the regularization coefficient plays an important role, which requires people to manually determine the size before classification and recognition. However, there is no effective parameter selection method at present. In most cases, people use trial and error method to select the size of the regular coefficient.
(3) Because the ELM has the defect of randomly giving the left weight and the hidden layer threshold, the regression model is prone to have low generalization performance and poor stability, which is crucial to classification problems.

Aiming at the shortcomings of the above ELM theory, this article takes the functional neuron (FN) model (Castillo, 1998; Guo et al., 2019) as the basic unit, intends to use the functional equation solving theory to guide the modeling process of extreme learning machine, and proposes a new type of functional extreme learning machine (FELM) theory. The functional neurons of the learning machine are not fixed, and they are usually linear combinations of linearly independent base functions. In FELM, network learning can be achieved by adjusting the coefficients of base functions in neurons. For the parameters (coefficients) selection method, one simple, iterative-free and high-precision parameter fast learning algorithm is proposed. Finally, through simulation experiments on the regression problems of real standard test data sets, compared with the traditional extreme learning machine (ELM) and support vector regression (SVR), the approximation ability, parameter learning speed, generalization performance and stability of the proposed FELM are experimentally tested.

The rest of the article is organized as follows: in Section “2. Functional extreme learning machine (FELM)”, we describe the FELM modeling theory, parameter learning algorithm and the feasibility analysis of the modeling theory in detail. Section “3. Experimental results and analysis” conducts regression experiments to evaluate the performance of the proposed technology. Finally, we summarize and future work in Section “4. Conclusions and future works”.

## 2. Functional extreme learning machine (FELM)

**FIGURE 1:**
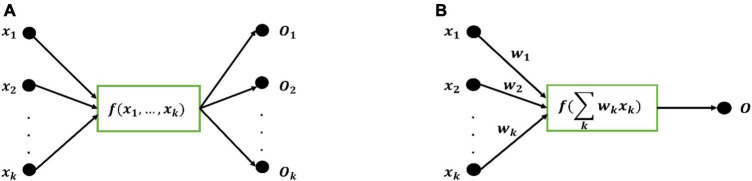
**(A)** Functional neuron model. **(B)** M-P neuron model.

Taking the FN model as the basic unit, a kind of learning machine with better performance is designed based on the functional equation solving theory, which is called Functional Extreme Learning Machine (FELM). The model is different from the traditional extreme learning machine. The type and quantity of hidden layer activation function in the structure of FELM are not fixed and can be adjusted. Figure 1A is the functional neuron model, and Figure 1B is the M-P neuron model. Comparing Figure 1A and Figure 1B, it can be seen that compared with artificial neurons, functional neurons lack the weight information on the connection line and can have multiple outputs. The output of functional neurons is:


(1) $$\left\{O_{1},O_{2},\ldots ,O_{k}\right\}=f\,\left(x_{1},x_{2},\ldots ,x_{k}\right).$$

Functional neuron *f* can be a linear combination of arbitrary nonlinear correlation base functions:


(2) $$f\,\left(x_{1},x_{2},\ldots ,x_{k}\right)=\sum_{j=1}^{n}a_{j}\,\varphi_{j}\,\left(x_{1},x_{2},\ldots ,x_{k}\right).$$

where {*φ*_*j*_(*x*_1_, *x*_2_, …, *x*_*k*_) | *j* = 1,2,…,*n*} is any given base functions, which can be learned. According to specific problems and data, different functions can be selected, such as trigonometric basis function and Fourier basis function; {*a*_*j*_|*j* = 1, 2, …, *n*} is a parameter set, which can also be learned. It can be used to infinitely approximate the expected accuracy of functional neuron function *f*(*x*_1_, *x*_2_, …, *x*_*k*_).

### 2.1. FELM model

With functional neuron as the basic unit (Figure 1A), the definition of general FELM is established:

**Definition 1:** Any FELM is a binary ordered pair: *FELM* = < *X*, *U* >, where *X* is a node set, *U* = { < *Y*_*i*_, *F*_*i*_, *Z*_*i*_ > |*i* = 1, 2, …, *n*} is a functional neuron set on the node set *X* and satisfies: For any node *X*_*i*_ ∈ *X*, it is an input node or an output node and at least one functional neuron belongs to the node set *U*.

**FIGURE 2:**
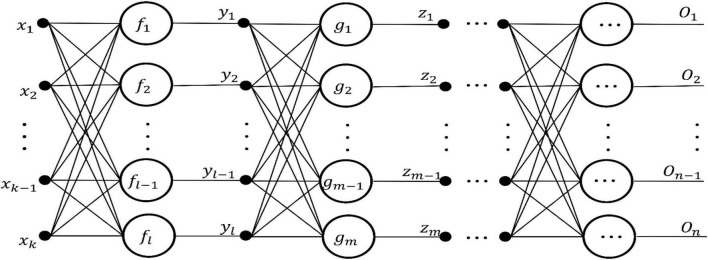
Topological structure of general FELM.

According to the definition of FELM, the components of general FELM include:

(1) Several layers of storage units: one layer input unit; one layer output unit; some intermediate units are used to store the information generated by functional neurons in the intermediate layer; all are represented by solid circles with corresponding names (i.e., {*x*_*i*_, *y*_*i*_, *z*_*i*_, …} in Figure 2).

(2) One layer or several layers of processing units: A processing unit is a functional neuron, which handles a set of input values from the previous layer of functional neurons or input units, and provides a set of input data for the next layer of functional neurons or output units. (i.e., {*f*_*i*_, *g*_*i*_, …} in Figure 2).
(3) Directional connection lines: it connects the storage unit and the processing unit, and the arrow indicates the flow direction of information.

All these elements together constitute the structure of the FELM, and determine the generalization ability of the FELM.

We based on the above definition and components of FELM, it is easy to design a general FELM. The network topology is shown in Figure 2:

The output expression of FELM in Figure 2 is:


(3) $$\left\{ \begin{array}{c} y_{1}=f_{1}\,\left(x_{1},x_{2},\ldots ,x_{k}\right),\\ y_{2}=f_{2}\,\left(x_{1},x_{2},\ldots ,x_{k}\right),\\ \vdots \\ z_{1}=g_{1}\,\left(y_{1},y_{2},\ldots ,y_{l}\right),\\ z_{2}=g_{2}\,\left(y_{1},y_{2},\ldots ,y_{l}\right),\\ \vdots \\ z_{m}=g_{m}\,\left(y_{1},y_{2},\ldots ,y_{l}\right),\\ \vdots \end{array}\right.$$

The Figure 2 shows the general FELM model, and the output expression (3) of the network is essentially a functional equation group. In turn, any functional equations can draw the corresponding functional learning machine. Therefore, it is concluded that any FELM establishes a one-to-one correspondence with the functional equation (group). Based on the correspondence between FELM and functional equation (group), the functional equation theory is used to guide the modeling process of FELM. The steps are as follows:

**Step 1**. Based on the characteristics of the undetermined problem and the definition of FELM, the initial FELM model for solving the undetermined problem is established.
**Step 2.** Obtain the output expression of the initial FELM; this expression corresponds to a functional equation group;
**Step 3**. Using the method of solving functional equations, the general solution expression is given.
**Step 4.** Based on the general solution expression of functional equations, the corresponding FELM is redrawn by using its one-to-one correspondence with FELM.
**Step 5.** Output the simplified FELM.

In this way, according to the above modeling steps of FELM, any type of FELM can be drawn, and the model establishes one-to-one correspondence with functional equation (group). Moreover, the functional equations are used to simplify and obtain any optimal FELM. The theoretical basis of the definition is based on the mathematical model of “binary ordered pairs” in discrete mathematics. Its physical meaning is similar to the layout structure of a printed circuit board (PCB). In the practical application of FELM modeling theory, based on the characteristics and data of the problem to be solved, the FELM (initial structure) of any problem to be solved can be obtained according to the above definition of general FELM and the theoretical guidance of modeling process by solving functional equation.

Based on the definition and constituent elements of FELM, any type of FELM can be drawn, and one-to-one correspondence with functional equation (group) can be established. Therefore, using functional equation solving theory to guide the design process of FELM is supported by mathematical theory, which is correct and easy to operate. The unique structure of FELM fundamentally overcomes the shortcomings of the current extreme learning machine that the weights randomly determined by hidden layer neurons have a great impact on the classification performance of the network, and the number of hidden layer neurons cannot be obtained by an effective algorithm.

### 2.2. FELM learning algorithm

**FIGURE 3:**
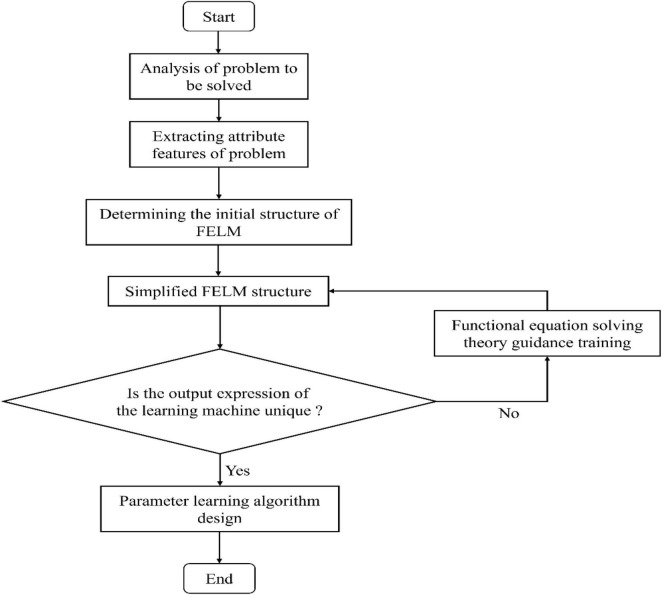
General FELM learning process diagram.

The FELM is based on the problem-driven modeling, without weight and threshold concepts. Its learning essence is to learn the network structure and parameters. Aiming at the parameter (coefficient) selection method, based on the parameter error cost function evaluation criterion, a simple, no iteration and high precision fast parameter learning algorithm is designed by using the theory of linear equations. The learning process of FELM is shown in Figure 3.

The learning process of FELM in Figure 5 is illustrated by a specific example:

Set a disease *d* with three basic symptoms: *x*: fever, *y*: dry cough; *z*: fatigue. How to build a FELM to implement its prediction so that: *d* = *D*(*x*, *y*, *z*).

**FIGURE 4:**
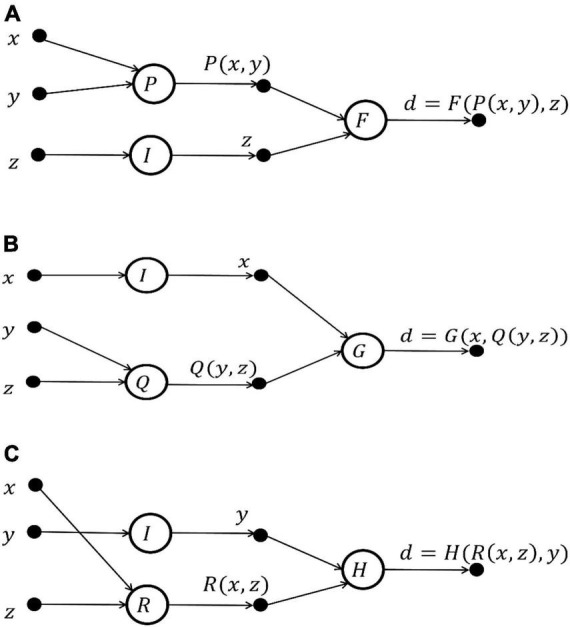
Initial structure of FELM for predicting disease *d* = *D*(*x*, *y*, *z*). **(A)** Diagnostic order: *x* → *y* → *z*. **(B)** Diagnostic order: *y* → *z* → *x*. **(C)** Diagnostic order: *x* → *z* → *y*.

(1) *Determine the initial structure of FELM.* According to the knowledge and information (known data, prior knowledge of the problem and some characteristics of the function, etc.) of the problem, the initial structure of FELM is designed. In the process of diagnosing a disease with three characteristics, the order of symptoms asked by doctors is different, and three cases of the initial structure are shown as in Figures 4A–C:

(2) *Simplifying the initial structure of FELM*. Since each initial network structure corresponds to a functional equation group, the FELMs equivalent to the initial network structure is found by using the characteristics of the solution of the functional equation, and the simple and optimal FELM equivalent to the initial network structure is selected.

The above examples in Figure 4 are essentially independent of the diagnostic order, which meet functional equation.


(4) d=D⁢(x,y,z)=F⁢(P⁢(x,y),z)=G⁢(Q⁢(y,z),x)

=H⁢(R⁢(x,z),y).

The general solution of Eq. 4 is:


(5) d=D⁢(x,y,z)=k⁢[p⁢(x)+q⁢(y)+r⁢(z)].

**FIGURE 5:**
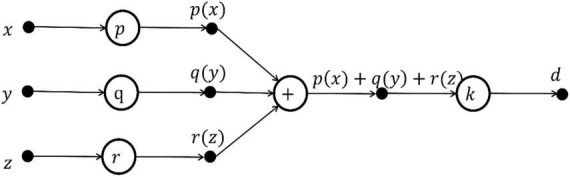
The FELM structure equivalent to Figure 4.

The FELM equivalent to functional Eq. 5 is shown in Figure 5.

In this way, the design network can be simplified by using the solution theory of functional equations, and the equivalent, a simple and optimal FELM can be obtained.

(3) *Uniqueness of output expression of functional extreme machine*. Before FELM learning, ensure the uniqueness of output expression. It is proved theoretically that for a given FELM, under the same initial conditions, the FELM has the same output value for any input value.

The above example is still used to prove the equivalence of FELMs in Figures 4, 5. It is assumed that there are two functional neuron function sets: {*k*_1_, *p*_1_, *q*_1_, *r*_1_} and {*k*_2_, *p*_2_, *q*_2_, *r*_2_}, so that


(6) $$k_{1}\,\left[p_{1}+q_{1}+r_{1}\right]=k_{2}\,\left[p_{2}+q_{2}+r_{2}\right],$$

For any variable *x, y, z*, let $k_{2}\,\left(u\right)=k_{1}\,\left(\frac{u-b-c-d}{a}\right)$, the solution of the functional equation is:


(7) $$p_{2}\,\left(x\right)=a\,p_{1}\,\left(x\right)+b;q_{2}\,\left(y\right)=a\,q_{1}\,\left(y\right)+c;r_{2}\,\left(z\right)=a\,r_{1}\,\left(z\right)+d.$$

Such uniqueness is proved.

**FIGURE 6:**
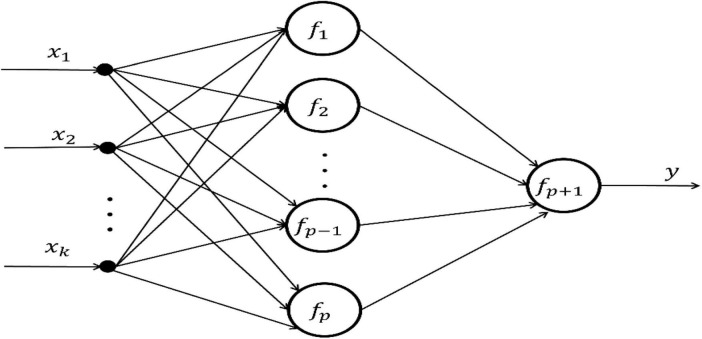
Multi-input single-output single-hidden layer FELM.

(4) *Parameter learning algorithm design for FELM*. In the general FELM in Figure 4, a multi-input single-output single-hidden layer FELM is selected as an example. Its network structure is shown in Figure 6:

Let input *X* = [*x*_1_, *x*_2_, …, *x*_*k*_] and output *y*; each neuron function in the hidden layer *f*_*i*_, *i* = 1, 2, …, *p* is a linear combination of any nonlinear correlation base functions, that is,


(8) $$f_{i}\,\left(x_{1},x_{2},\ldots ,x_{k}\right)=\sum_{j=1}^{m}a_{i\,j}\,\varphi_{i\,j}\,\left(x_{1},x_{2},\ldots ,x_{k}\right).$$

where *m* is the number of functional neuron base functions. For the convenience of matrix representation. Let *s*_*i*_ = *X* ● ω_*i*_, ω_*i*_ = [1, 1, …, 1]^*T*^. Then Eq. 8 can be written:


(9) $$f_{i}\,\left(s_{i}\right)=\sum_{j=1}^{m}a_{i\,j}\,\varphi_{i\,j}\,\left(s_{i}\right).$$

when the function of output neuron has inverse function, it can also be expressed as a linear combination of base functions:


(10) $$f_{p+1}^{-1}\,\left(x\right)=-\sum_{j=1}^{m}a_{j}\,\varphi_{j}\,\left(x\right).$$

The output of the FELM in Figure 6 is:


(11) $$y=f_{p+1}\,\left(\sum_{i=1}^{p}\sum_{j=1}^{m}a_{i\,j}\,\varphi_{i\,j}\,\left(s_{i}\right)\right).$$

Let the sample data be (*X*_*r*_, *y*_*r*_) and the error cost is:


(12) $$e_{r}=y_{r}-f_{p+1}\,\left(\sum_{i=1}^{p}\sum_{j=1}^{m}a_{i\,j}\,\varphi_{i\,j}\,\left(s_{r\,i}\right)\right).$$

where *s*_*ri*_ = *X*_*r*_ω_*i*_.

If there are *n* groups of sample data, the sum of squares of error of the FELM model is:


(13) $$E=\sum_{r=1}^{n}{\left(y_{r}-f_{p+1}\,\left(\sum_{i=1}^{p}\sum_{j=1}^{m}a_{i\,j}\,\varphi_{i\,j}\,\left(s_{r\,i}\right)\right)\right)}^{2}.$$

By changing the value of the base function coefficient *a*_*ij*_, *E* is minimized.

If *f*_*p+1*_ is a reversible function, the error sum of the FELM model can be expressed as:


(14) $$E=\sum_{r=1}^{n}{\left(\sum_{i=1}^{p+1}\sum_{j=1}^{m}\begin{array}{c} a_{i\,j}\,\varphi_{i\,j}\,\left(s_{r\,i}\right) \end{array}\right)}^{2}.$$

where *s*_*r*,*p* + 1_ = *y*_*r*_.

#### 2.2.1. Parameter learning algorithm

The optimal value of parameter coefficient *a*_*ij*_ can be obtained by solving (15):


(15) $$\frac{\partial E}{\partial a_{t\,s}}=2\,\sum_{r=1}^{n}\begin{array}{c} \left(\sum_{i=1}^{p+1}\sum_{j=1}^{m}a_{i\,j}\,\varphi_{i\,j}\,\left(s_{r\,i}\right)\right)\,\varphi_{t\,s}\,\left(s_{r\,t}\right) \end{array}$$

$$=2\,\sum_{i=1}^{p+1}\sum_{j=1}^{m}\left(\sum_{r=1}^{n}\varphi_{t\,s}\,\left(s_{r\,t}\right)\,\varphi_{i\,j}\,\left(s_{r\,i}\right)\right)\,a_{i\,j}=0.$$

where *t* = 1, 2, …, *p* + 1; *s* = 1, 2, …, *m*.

The Eq. 15 is a linear equation group, which is easy to solve. Expand Eq. 15, such as Eq. 16:


(16) $$\left\{ \begin{array}{c} \frac{\partial E}{\partial a_{11}}=2\,e_{1}\,\varphi_{11}\,\left(s_{11}\right)+2\,e_{2}\,\varphi_{11}\,\left(s_{21}\right)\\ +\ldots +2\,e_{n}\,\varphi_{11}\,\left(s_{n\,1}\right)=0,\\ \frac{\partial E}{\partial a_{12}}=2\,e_{1}\,\varphi_{12}\,\left(s_{11}\right)+2\,e_{2}\,\varphi_{12}\,\left(s_{21}\right)\\ +\ldots +2\,e_{n}\,\varphi_{12}\,\left(s_{n\,1}\right)=0,\\ \vdots \\ \frac{\partial E}{\partial a_{1\,m}}=2\,e_{1}\,\varphi_{1\,m}\,\left(s_{11}\right)+2\,e_{2}\,\varphi_{1\,m}\,\left(s_{21}\right)\\ +\ldots +2\,e_{n}\,\varphi_{1\,m}\,\left(s_{n\,1}\right)=0,\\ \frac{\partial E}{\partial a_{21}}=2\,e_{1}\,\varphi_{21}\,\left(s_{12}\right)+2\,e_{2}\,\varphi_{21}\,\left(s_{22}\right)\\ +\ldots +2\,e_{n}\,\varphi_{21}\,\left(s_{n\,2}\right)=0,\\ \vdots \\ \frac{\partial E}{\partial a_{2\,m}}=2\,e_{1}\,\varphi_{2\,m}\,\left(s_{12}\right)+2\,e_{2}\,\varphi_{2\,m}\,\left(s_{22}\right)\\ +\ldots +2\,e_{n}\,\varphi_{2\,m}\,\left(s_{n\,2}\right)=0,\\ \vdots \\ \frac{\partial E}{\partial a_{p+1,1}}=2\,e_{1}\,\varphi_{p+1,1}\,\left(s_{1,p+1}\right)+2\,e_{2}\,\varphi_{p+1,1}\,\left(s_{2,p+1}\right)+\\ \ldots +2\,e_{n}\,\varphi_{p+1,1}\,\left(s_{n,p+1}\right)=0,\\ \vdots \\ \frac{\partial E}{\partial a_{p+1,m}}=2\,e_{1}\,\varphi_{p+1,m}\,\left(s_{1,p+1}\right)+2\,e_{2}\,\varphi_{p+1,m}\,\left(s_{2,p+1}\right)\\ +\ldots +2\,e_{n}\,\varphi_{p+1,m}\,\left(s_{n,p+1}\right)=0. \end{array}\right.$$

$$A=(\begin{array}{ccc} \varphi_{11}\,\left(s_{11}\right)\;\,\varphi_{11}\,\left(s_{21}\right)\;\,\cdots & & \\ \varphi_{12}\,\left(s_{11}\right)\;\,\varphi_{12}\,\left(s_{21}\right)\;\,\cdots & & \\ \vdots \,\ddots & & \\ \varphi_{1\,m}\,\left(s_{11}\right)\;\,\varphi_{1\,m}\,\left(s_{21}\right)\;\,\cdots & & \\ \varphi_{21}\,\left(s_{12}\right)\;\,\varphi_{21}\,\left(s_{22}\right)\;\,\cdots & & \\ \vdots \,\ddots & & \\ \varphi_{2\,m}\,\left(s_{12}\right)\;\,\varphi_{2\,m}\,\left(s_{22}\right)\,\;\cdots & & \\ \vdots \,\ddots & & \\ \varphi_{p+1,1}\,\left(s_{1,p+1}\right)\,\varphi_{p+1,1}\,\left(s_{2,p+1}\right)\,\;\cdots & & \\ \vdots \,\ddots & & \\ \varphi_{p+1,m}\,\left(s_{1,p+1}\right)\,\varphi_{p+1,m}\,\left(s_{2,p+1}\right)\;\,\cdots & & \end{array}$$

$$\begin{array}{cc} \varphi_{11}\,\left(s_{n-1,1}\right)\,\;\varphi_{11}\,\left(s_{n\,1}\right) & \\ \varphi_{12}\,\left(s_{n-1,1}\right)\,\;\varphi_{12}\,\left(s_{n\,1}\right) & \\ \;\vdots & \\ \varphi_{1\,m}\,\left(s_{n-1,1}\right)\,\;\varphi_{1\,m}\,\left(s_{n\,1}\right) & \\ \varphi_{21}\,\left(s_{n-1,2}\right)\,\;\varphi_{21}\,\left(s_{n\,2}\right) & \\ \;\vdots & \\ \varphi_{2\,m}\,\left(s_{n-1,2}\right)\,\;\varphi_{2\,m}\,\left(s_{n\,2}\right) & \\ \;\vdots & \\ \varphi_{p+1,1}\,\left(s_{n-1,p+1}\right)\,\;\varphi_{p+1,1}\,\left(s_{n,p+1}\right) & \\ \;\vdots & \\ \varphi_{p+1,m}\,\left(s_{n-1,p+1}\right)\;\,\varphi_{p+1,m}\,\left(s_{n,p+1}\right) & \end{array}),B=[\begin{array}{c} e_{1}\\ e_{2}\\ \vdots \\ e_{n} \end{array}].$$

In matrix form, Eq. 16 can be written as:


(17) 2⁢A⁢B=0

Let *P* = [*a*_11_, *a*_12_, ⋯, *a*_1*m*_, *a*_21_, ⋯, *a*_2*m*_, ⋯, *a*_*p*+1,1_, ⋯, *a*_*p*+1,*m*_]. So *B* can be written as $B=\left[\begin{array}{c} e_{1}\\ e_{2}\\ \vdots \\ e_{n} \end{array}\right]=A^{T}P^{T}$. So Eq. 17 is:


(18) $$\begin{array}{c} 2\,A\,A^{T}\,P^{T}=0. \end{array}$$

In Eq. 18, the vector *P* is the desired parameter coefficients, but it will not be unique. In order to solve this problem, initial constraint conditions need to be given. Suppose the given constraints are as follows.


(19) $$\begin{array}{c} f_{i}\,\left(x_{0}\right)=\sum_{j=1}^{m}a_{j}\,\varphi_{j}\,\left(s_{0}\right)=\beta_{i},i=1,2,\ldots ,p+1 \end{array}$$

where s_0_ = *X*_0_*ω*_*i*_, *i* = 1,2,…, *P* and *s*_0_ = *y*_0_, *i* = *P* + 1; *β*_*i*_ is any real constant. Therefore, by using the Lagrange multipliers technique, the following auxiliary function can be established.


(20) $$\begin{array}{c} E_{a\,d\,d}=\sum_{r=1}^{n}{\left(\sum_{i=1}^{p+1}\sum_{j=1}^{m}\begin{array}{c} a_{i\,j}\,\varphi_{i\,j}\,\left(s_{r\,i}\right) \end{array}\right)}^{2}+\sum_{s=1}^{p+1}\theta_{s}\,\left(\sum_{j=1}^{m}a_{s\,j}\,\varphi_{s\,j}\,\left(s_{0}\right)-\beta_{s}\right). \end{array}$$

The minimum model error sum of squares corresponds to.


(21) $$\begin{array}{c} \frac{\partial E_{a\,d\,d}}{\partial a_{t\,s}}=2\,\sum_{r=1}^{n}\begin{array}{c} \left(\sum_{i=1}^{p+1}\sum_{j=1}^{m}a_{i\,j}\,\varphi_{i\,j}\,\left(s_{r\,i}\right)\right)\,\varphi_{t\,s}\,\left(s_{r\,t}\right) \end{array}+\theta_{t}\,\varphi_{t\,s}\,\left(s_{0}\right)\\ =2\,\sum_{i=1}^{p+1}\sum_{j=1}^{m}\left(\sum_{r=1}^{n}\varphi_{t\,s}\,\left(s_{r\,t}\right)\,\varphi_{i\,j}\,\left(s_{r\,i}\right)\right)\,a_{i\,j}+\theta_{t}\,\varphi_{t\,s}\,\left(s_{0}\right)=0.\\ \begin{array}{c} \frac{\partial E_{a\,d\,d}}{\partial \theta_{t}}=\sum_{j=1}^{m}a_{t\,j}\,\varphi_{t\,i}\,\left(s_{0}\right)-\beta_{t}=0. \end{array} \end{array}$$

where *t* = 1, 2, …, *p* + 1; *s* = 1, 2, …, *m*.

Let


$$\varphi_{0}=\left[\begin{array}{ccccc} \varphi_{11}\left(s_{0}\right) & 0 & 0 & \cdots & 0\\ \vdots & & & & \\ \varphi_{1m}\left(s_{0}\right) & 0 & \cdots & 0 & 0\\ 0 & \varphi_{21}\left(s_{0}\right) & 0 & \cdots & 0\\ \vdots & & & & \\ 0 & 0 & \cdots & 0 & \varphi_{p+1}\left(s_{0}\right) \end{array}\right],$$

$$\theta =\left[\begin{array}{c} \theta_{1}\\ \theta_{2}\\ \vdots \\ \theta_{p+1} \end{array}\right],C=\left[\begin{array}{c} 0\\ \vdots \\ 0\\ \beta_{1}\\ \vdots \\ \beta_{p+1} \end{array}\right].$$

In matrix form, Eq. 21 can be written as:


(22) $$\begin{array}{c} \left[\begin{array}{cc} 2\,A\,A^{T} & \varphi_{0}\\ \varphi_{0}^{T} & 0 \end{array}\right]\,\left[\begin{array}{c} P^{T}\\ \theta \end{array}\right]=C. \end{array}$$

It is very simple to use Eq. 22 to solve the parameters. This parameter learning algorithm is simple, iterative-free, and has good approximation effect.

#### 2.2.2. FELM parameter learning algorithm analysis

The FELM is based on problem-driven, learning network structure and parameters. Its each step in the learning process is operable and realizable, and the learning process is suitable for any FELM. At the same time, the theoretical basis and mathematical derivation of learning algorithms of FELM are given. The parameter learning algorithm is simple, no iteration and high precision, which is convenient for engineers to use.

The learning process of FELM is completely different from that of the ELM, for its structure have no weight values and the threshold value of neurons. In the ELM, the input layer weights and hidden neuron thresholds of the network are randomly selected, but people can only choose the size of the regularization coefficient by trial and error method, because there is no effective parameter selection method. The characteristics of FELM structure and the process of parameter learning make the problem fundamentally solved. Based on some simple examples, the above shows the learning process of the structure and parameters of the FELM, and its research ideas can be extended to general situations.

## 3. Experimental results and analysis

In this section, the performance of the proposed FELM is compared with feedforward neural network algorithms such as the ELM and support vector regression (SVR) on approximating two artificial datasets and 16 benchmark real problems. For comparison, three variant algorithms of ELM (OP-ELM) (Miche et al., 2010), inverse-free ELM (Li et al., 2016), OS-RELM (Shao and Er, 2016) and the variant algorithm of SVR (LSSVR) are also added. Simulations of all algorithms are performed in the MATLAB 2019b environment running on an 11th Gen Intel(R) Core(TM) i5-11320H @ 3.20GHz and 16GB RAM.

The SVR, LSSVR, ELM, and OP-ELM source codes used in this experiment were downloaded from www.csie.ntu.edu.tw/cjlin/libsvm/,www.esat.kuleuven.be/sista/lssvmlab/;www.ntu.edu.sg/home/egbhuang/; and www.cis.hut.fi/projects/tsp/index.php?page=OPELM, respectively. We use the radial basis function as the kernel function for SVR and LSSVR. In SVR, two parameters are mainly optimized. For each problem we use different combinations of the cost parameter *C* and the kernel parameter γ to estimate the generalized accuracy (Huang and Zhao, 2018): *C* = [2^12^, 2^11^, …, 2^−1^, 2^−2^] and γ = [2^4^, 2^3^, …, 2^−9^, 2^−10^] (Huang et al., 2006). Therefore, on SVR, for each problem we try 15 × 15 = 225 (*C*,γ) parameter combinations, 50 trials for each combination, then calculate the root mean square error (RMSE) of the 50 results of the combination, take the combination with the smallest root mean square error among the 225 combinations as the best parameter combination, and the parameter optimization process of LSSVR is the same. LSSVR mainly optimizes the regularization parameter (*C*) and the kernel parameter (*k*_*p*_), and the adopted ranges are the same as *C* and γ of SVR, respectively. The activation functions of ELM and its variants and the base functions of FELM will be set according to the following specific problems to be solved.

### 3.1. Artificial datasets

#### 3.1.1. Artificial case 1: *f*_1_ (*x*) = *x* cos(4*x*)

In the interval [−1.0, 1.5], 101 training samples (*x*_i_, *f*_*i*_) were obtained by sampling at 0.025 intervals. In addition, with 0.025 as the interval, nine data points are obtained in the unlearned interval [1.505, 1.705] as the prediction points. In this example, FELM uses the base functions: {sin (*x*), sin (2*x*), sin (3*x*), sin (4*x*), sin (5*x*), sin (6*x*)}. The optimal combination parameter of SVR is (*C*, γ) = (2^12^, 2^0^), the optimal combination parameter of LSSVR: (*C*, *k*_*p*_) = (2^12^, 2^−10^). ELM, OP-ELM and inverse-free ELM use the sig function as the activation function, and the activation function of OS-RELM is triangular basis function, because the commonly used sig function cannot obtain a feasible solution to this problem in a reasonable time. Trigonometrically-Activated Fourier Neural Networks (Zhang et al., 2009a) (hereinafter referred to as TAFNN) will also be added for performance comparison. The definition error of function as follows.


(23) $$\begin{array}{c} E=\frac{1}{2}\,\sum_{t=1}^{N}e_{t}^{2}=\frac{1}{2}\,\sum_{t=1}^{N}{\left(e\,x\,p\,e\,c\,t_{t}-p\,r\,e\,d\,i\,c\,t_{t}\right)}^{2} \end{array}$$

where *expect*_*t*_ is the expected output, and *predict*_*t*_ represents the actual network output, *N* is the number of sample points. And *E*/*N* is the average error.

**FIGURE 7:**
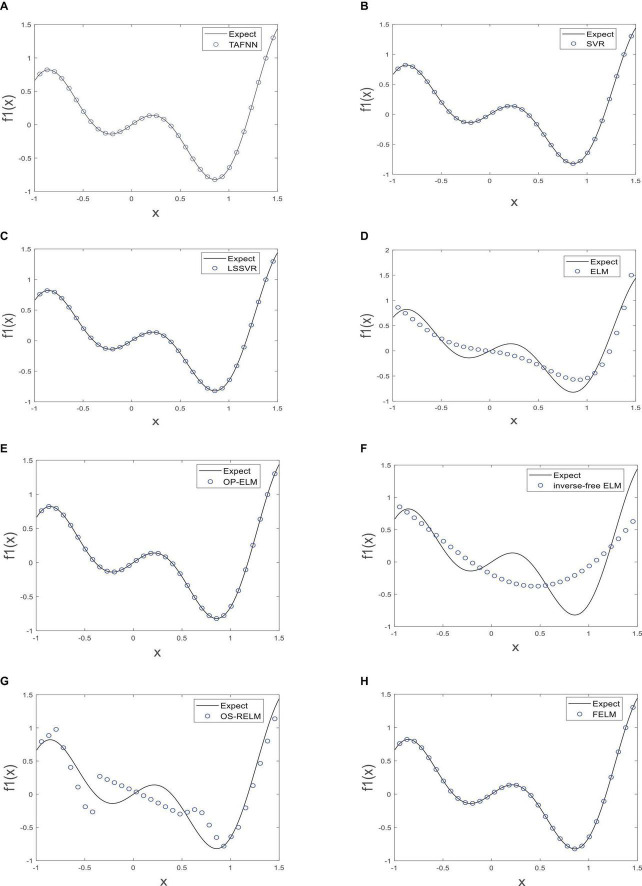
The test of each algorithm [**(A)** TAFNN, **(B)** SVR, **(C)** LSSVR, **(D)** ELM, **(E)** OP-ELM, **(F)** Inverse-free ELM, **(G)** OS-RELM, and **(H)** FELM] on *f*_1_(*x*).

**FIGURE 8:**
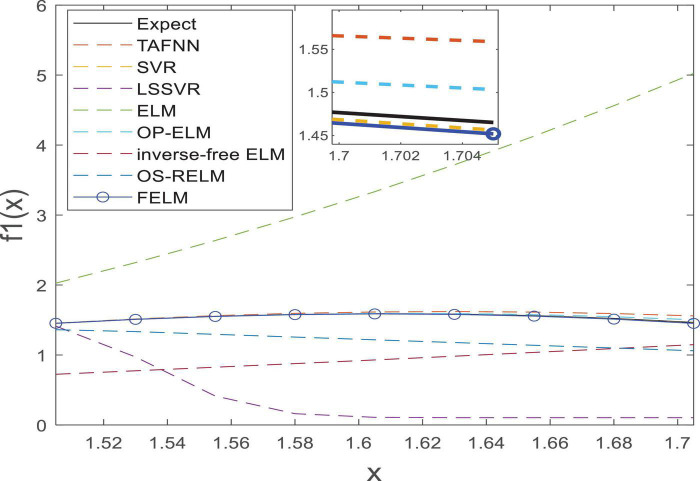
The prediction on *f*_1_(*x*).

As shown in Table 1, the parts in bold are the optimal data, FELM has a competitive advantage in training time, and the total training error and the average training error are less than 6 compared algorithms (especially hundreds of thousands of times smaller than ELM, inverse-free ELM and OS-RELM, Figure 7 shows that the training results obtained by the above three algorithms are not satisfactory), but slightly worse than OP-ELM. However, the training time of FELM is more than 200 times faster than OP-ELM, and its hidden layer has only 6 parameters, so its model complexity is much lower than OP-ELM. Figure 7 shows the testing of 8 algorithms. It can be seen that the testing results of FELM are good, and the error between its output and the target output is small. The testing results of ELM, inverse-free ELM and OS-RELM are different from the target output. In terms of prediction accuracy, FELM obtains the smallest total prediction error and average prediction error which shows that it has good generalization performance. A more intuitive comparison of predictions is shown in Figure 8. Therefore, compared with other comparison algorithms, FELM can obtain the highest prediction accuracy in the shortest time under the smallest network model.

**TABLE 1:** Comparison of simulation and prediction on the *f*_1_(*x*).

Algorithms	Train time (s)	*E* (training)	*E*/*N* (training)	Model^a^	*E*/*N* (testing)	*E* (prediction)	*E*/*N* (prediction)
TAFNN (Zhang et al., 2009a)	5.0558E-04	1.2441E-05	1.2318E-07	6	1.3355E-07	9.3008E-03	1.0334E-03
SVR	3.0791E+00	2.1591E-06	2.1377E-08	37	1.8650E-08	8.5675E-04	9.5195E-05
LSSVR	4.5523E-03	3.1328E-07	3.1017E-09	101	8.7980E-06	6.9867E+00	7.7630E-01
ELM	1.0739E-03	1.5963E+00	1.5805E-02	6	1.6132E-02	2.0097E+01	2.2330E+00
OP-ELM	1.0445E-01	**1.0288E-08**	**1.0186E-10**	87/25*	**1.0350E-10**	1.1865E-03	1.3184E-04
Inverse-free ELM	1.8147E-03	5.6601E+00	5.6040E-02	6	5.6675E-02	1.9901E+00	2.2112E-01
OS-RELM	4.5930E-04	2.2948E+00	2.2721E-02	6	2.2719E-02	5.1731E-01	5.7479E-02
FELM	3.4860E-04	5.4485E-08	5.3945E-10	6	5.9727E-10	1.6918E-04	1.8798E-05

^a^The number of hidden nodes of TAFNN, ELM and its three variant algorithms, the number of support vectors of SVR and LSSVR, and the number of hidden layer parameters of FELM.

*Maximum number of neurons given before training/optimal number of neurons after training.

#### 3.1.2. Artificial case 2: *f*_2_ (*x*) = *e^x^* cos (6π*x*) /3 sin (*x*)

In the interval [−1, 1], 201 training samples are obtained at 0.01 sampling interval. The following simulation is carried out to determine the optimal number of hidden neurons corresponding to four different expectation average error(*E*_*expect*_ = 10^−2^, 10^−3^, 10^−4^, 10^−5^), the evaluation metric is Eq. 23. The FELM uses {1, *x*, *x*^2^, *x*^3^, …, *x*^24^} as the base functions in this example. For different expected mean errors, the best parameters of SVR (*C*, γ) = (2^−1^, 2^4^), (2^1^, 2^4^), (2^4^, 2^4^), (2^5^, 2^4^). The best parameters of LSSVR (*C*, *k*_*p*_) = (2^−2^, 2^−10^), (2^0^, 2^−10^), (2^2^, 2^−10^), (2^4^, 2^−10^). OP-ELM uses the commonly used activation function sig function, and the activation function of ELM, inverse-free ELM and OS-RELM is triangular basis function. The Legendre neural network (Zhang et al., 2009b) (hereinafter referred to as Legendre) will also be used for comparison.

**FIGURE 9:**
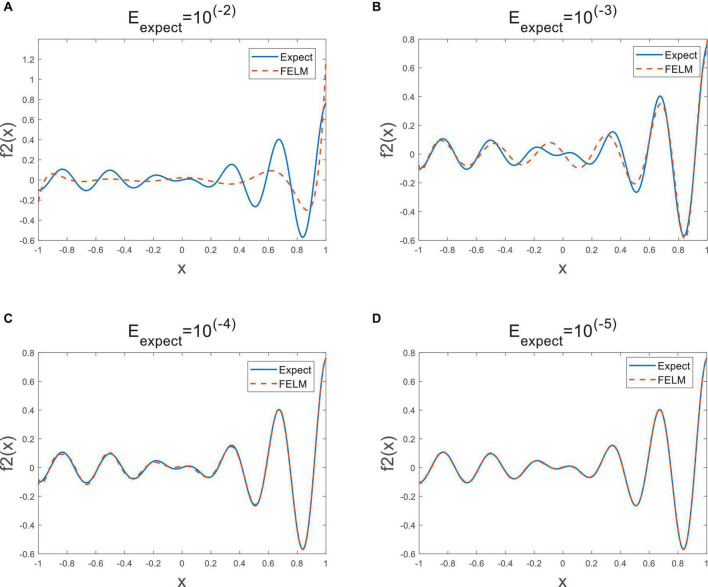
The approximation error [**(A–D)** 10^−2^∼10^−5^] of FELM.

As shown in Table 2, the parts in bold are the optimal data, except for the first *E*_exp *ect*_, under the other three *E*_exp *ect*_, the structural complexity of FELM is the lowest with the Legendre, because they use similar hidden layer functions. It can be seen from the table that the time required for the FELM optimization process is short, indicating that of its learning speed is fast. Table 2 shows that the higher the required precision, the network complexity of ELM, inverse-free ELM and OS-RELM increase exponentially, while the model complexity of SVR has always been relatively large, and the structural complexity of LSSVR remains unchanged because all training samples have been used all the time. In contrast to FELM, its structural complexity will not increase significantly due to high precision requirements, but will increase slowly. Figure 9 shows the approximation of FELM under four different required accuracies.

**TABLE 2:** Comparison of the simplest network structure and running time for approximating *f*_2_(*x*) at four precisions.

Algorithms	E_expect_							
	**10^−2^**		**10^−3^**		**10^−4^**		**10^−5^**	
	**Model^a^**	**Time**	**Model^a^**	**Time**	**Model^a^**	**Time**	**Model^a^**	**Time**
Legendre (Zhang et al., 2009b)	10	0.01113	17	0.01844	22	0.02531	24	0.02635
SVR	107	0.19271	111	0.81580	127	4.24594	120	8.34329
LSSVR	201	1.20236	201	0.31068	201	0.04934	201	0.03241
ELM	14	0.03886	32	0.09619	51	0.16112	115	0.45132
OP-ELM	**9**	0.08181	17	0.37463	32	0.25154	32	0.21841
inverse-free ELM	17	0.03072	43	0.02585	116	**0.13123**	366	1.28757
OS-RELM	10	0.00882	42	0.02152	96	0.08197	364	1.21366
FELM	10	**0.00585**	17	**0.01059**	22	0.01430	24	**0.01585**

^a^The minimum number of hidden nodes for Legendre and ELM that meet the accuracy requirements, the number of support vectors for SVR, and the minimum number of parameters for FELM.

### 3.2. Realistic regression problem

#### 3.2.1. Data sets and experimental settings

The 16 real benchmark datasets are selected because they cover various fields, and the data size and dimension are different. They are mainly obtained from the data archives of UCI Machine Learning (Asuncion and Newman, 2007) and StatLib (StatLib DataSets Archive, 2021). Table 3 lists the specifications of these datasets. In practical applications, the distribution of these datasets is unknown, and most datasets are not noise-free. For these 7 algorithms, 50 independent simulation trials are performed on each dataset, and the training and test data are randomly regenerated from their entire dataset, two-thirds training and one-third testing. Additionally, in our experiments, all inputs (attributes) and outputs (targets) are normalized to the range [−1, 1].

**TABLE 3:** Examples of actual regression.

Datasets	#Train	#Test	#Total	#Features
Abalone	2,784	1,393	4,177	8
Mpg	261	131	392	7
Autoprice	106	53	159	15
Balloon	1,334	667	2,001	2
Baskball	64	32	96	4
Cleveland	202	101	303	13
Cloud	72	36	108	9
Concrete CS	686	344	1,030	8
Diabetes	28	15	43	2
Housing	337	169	506	13
Machine CPU	139	70	209	6
Mg	923	462	1,385	6
Quake	1,452	726	2,178	3
Servo	111	56	167	4
Strike	416	209	625	6
Wisconsin B.C.	129	65	194	32

The ELM uses the sig function as the activation function, OP-ELM uses Gaussian kernel, and the proposed algorithm uses two different types of base functions: φ_1_ = {1, *x*, *x*^2^, *x*^3^} and φ_1_ = {sin(*x*), sin(2*x*), sin(3*x*)}, the rest of the comparison algorithms use the RBF kernel. The network complexity comparison of FELM, ELM and SVR is shown in Table 4, where FELM and ELM adopt the same network complexity on the same problem, it can be seen that in most cases, FELM is more compact than SVR. It should be noted that, for fairness, the network complexity of OP-ELM, inverse-free ELM and OS-RELM is also the same as ELM, and will not be repeated in the table. Finally, on the baskball, cloud and diabetes problems, the maximum number of neurons for OP-ELM is pre-specified as 62, 70 and 26, respectively, because they have fewer training sets, and the remaining data sets are used a maximum number of 100 neurons.

**TABLE 4:** Comparison of network complexity.

Algorithm	FELM	SVR		LSSVR		ELM
	**# nodes**	**(*C*, γ)**	**# SVs**	**(*C*, γ)**	**# SVs**	**# nodes**
Abalone	8	(2^11^,2^4^)	1,461.82	(2^12^,2^−10^)	2,784	8
Mpg	7	(2^8^,2^4^)	182.64	(2^12^,2^−7^)	261	7
Autoprice	15	(2^9^,2^3^)	105.38	(2^12^,2^−10^)	106	15
Balloon	2	(2^7^,2^4^)	690.18	(2^12^,2^−10^)	1,334	2
Baskball	4	(2^10^,2^0^)	58.08	(2^12^,2^−10^)	64	4
Cleveland	13	(2^11^,2^−4^)	197.92	(2^12^,2^−4^)	198	13
Cloud	9	(2^7^,2^2^)	71.94	(2^12^,2^−5^)	72	9
Concrete CS	8	(2^11^,2^4^)	527.82	(2^11^,2^−9^)	686	8
Diabetes	2	(2^11^,2^3^)	24.32	(2^12^,2^−10^)	28	2
Housing	13	(2^12^,2^−1^)	329.76	(2^12^,2^−6^)	337	13
Machine CPU	6	(2^7^,2^3^)	108.52	(2^12^,2^−7^)	139	6
Mg	6	(2^12^,2^4^)	530.38	(2^12^,2^−10^)	923	6
Quake	3	(2^11^,2^4^)	756.86	(2^12^,2^−10^)	1,452	3
Servo	4	(2^2^,2^4^)	77.56	(2^12^,2^−3^)	111	4
Strike	6	(2^12^,2^1^)	341.7	(2^12^,2^−9^)	416	6
Wisconsin B.C.	32	(2^11^,2^−5^)	128.28	(2^12^,2^0^)	129	32

#### 3.2.2. Evaluation criteria

On the above 13 benchmark regression problems, the evaluation criteria of FELM, ELM and SVR adopt root mean square error:


(24) $$R\,M\,S\,E=\sqrt{\frac{1}{N}\,\sum_{t=1}^{N}{\left(p\,r\,e\,d\,i\,c\,t\,e\,d_{t}-l\,a\,b\,e\,l_{t}\right)}^{2}}$$

#### 3.2.3. Evaluation and analysis of experimental results

**FIGURE 10:**
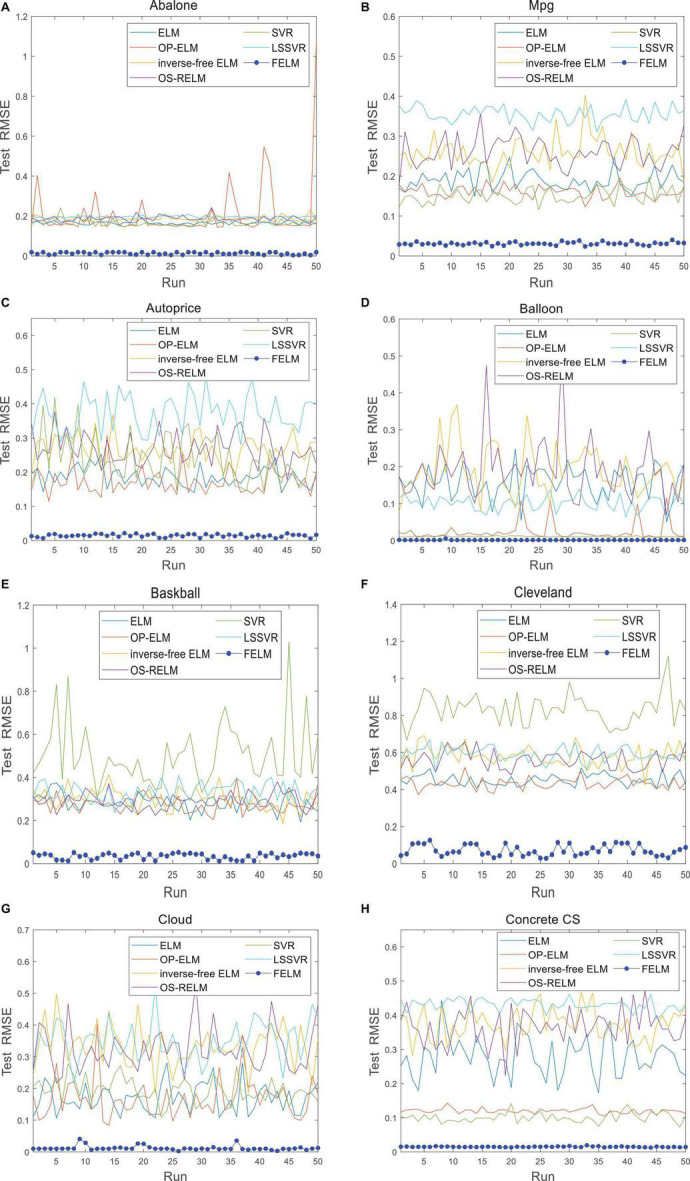
Comparison of test RMSE on the first 8 datasets when FELM uses φ_*1*_. **(A)** Abalone data set, **(B)** Mpg data set, **(C)** autoprice data set, **(D)** balloon data set, **(E**) baskball data set, **(F)** cleveland data set, **(G)** cloud data set, and **(H)** concrete CS data set.

**FIGURE 11:**
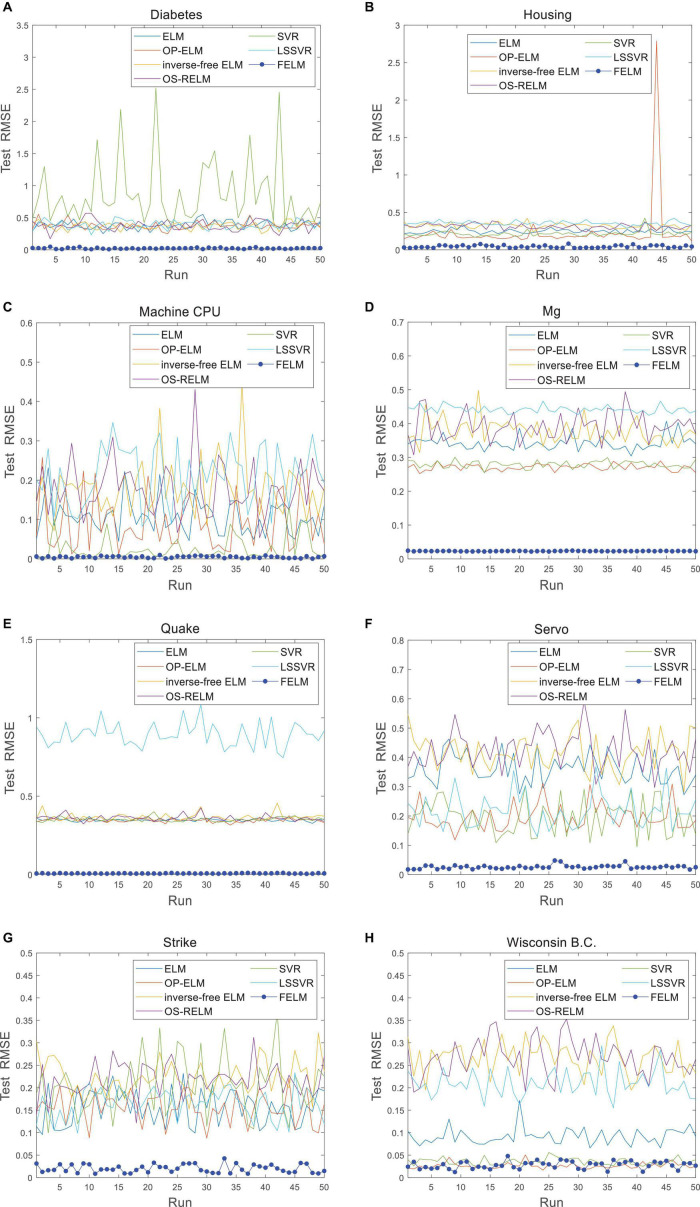
Comparison of test RMSE on the last 8 datasets when FELM uses *φ*_*1*_. **(A)** Diabetes data set, **(B)** housing data set, **(C)** machine CPU data set, **(D)** Mg data set, **(E)** quake data set, **(F)** servo data set, **(G)** strike data set, and **(H)** wisconsin B.C. data set.

**FIGURE 12:**
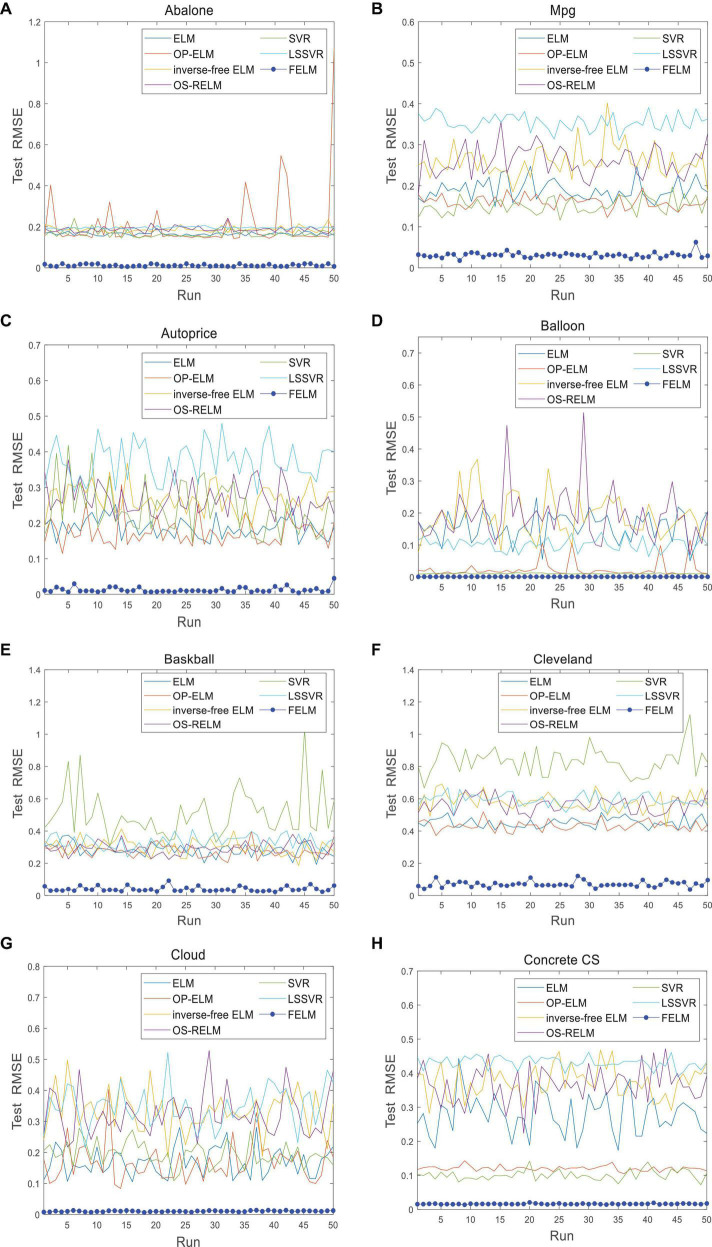
Comparison of test RMSE on the first 8 datasets when FELM uses *φ*_*2*_. **(A)** Abalone data set, **(B)** Mpg data set, **(C)** autoprice data set, **(D)** balloon data set, **(E**) baskball data set, **(F)** cleveland data set, **(G)** cloud data set, and **(H)** concrete CS data set.

**FIGURE 13:**
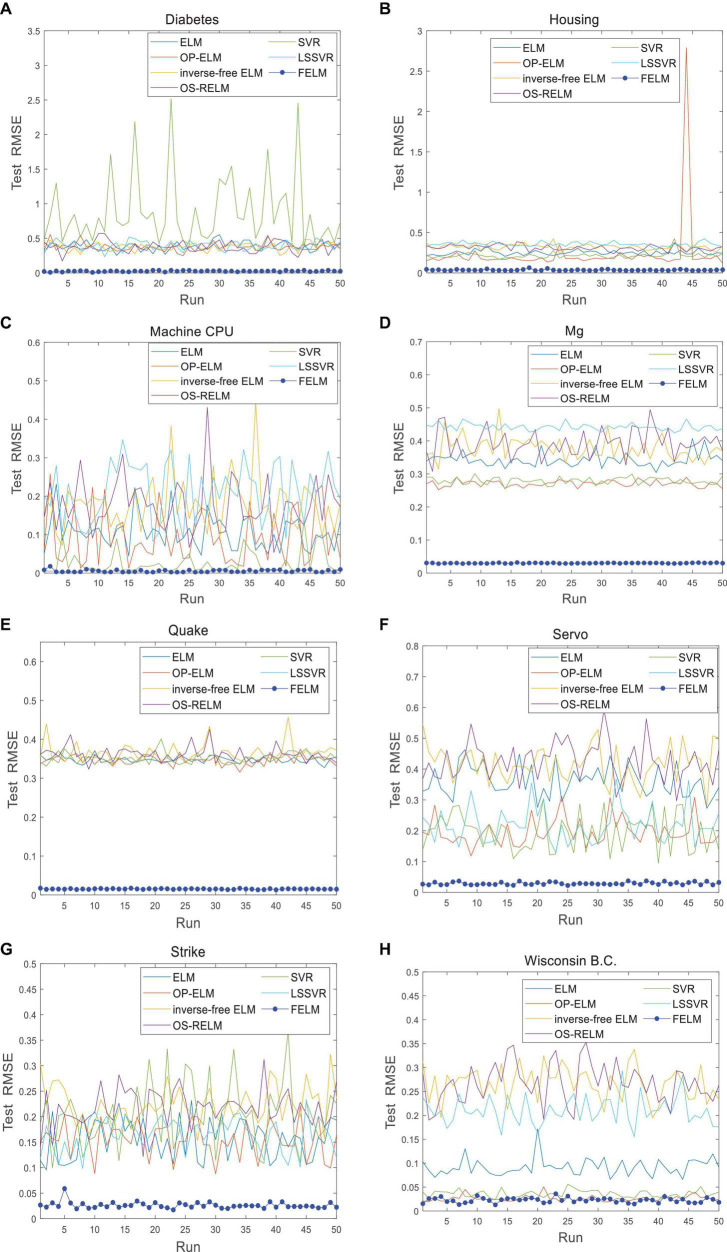
Comparison of test RMSE on the last 8 datasets when FELM uses *φ*_*2*_. **(A)** Diabetes data set, **(B)** housing data set, **(C)** machine CPU data set, **(D)** Mg data set, **(E)** quake data set, **(F)** servo data set, **(G)** strike data set, and **(H)** wisconsin B.C. data set.

The FELM is compared with other algorithms to test RMSE under two different types of base functions, and the winners are shown in Tables 5, 6 in bold. As can be seen from Tables 5, 6, FELM achieves higher generalization performance than the other 6 algorithms on all problems. Except on the Wisconsin B.C. problem, the average test RMSE of FELM is 1 order of magnitude higher than the other algorithms on the other 15 problems. It is worth noting that in Table 6, LSSVR achieves the best training results, but on most problems, its test and training RMSE are 3 orders of magnitude different, while the training and test RMSE of FELM are of the same order of magnitude or only one order of magnitude worse. Figures 10–13 shows that whether FELM uses φ_*1*_ or φ_*2*_, on Balloon, the curve of FELM and SVR are at the same level and below, which shows that although they obtain similar results, they are better than other algorithms. In Wisconsin B.C., The comparison curves of FELM, SVR and OP-ELM are also similar. But for other 14 problems, it can be seen from the figure that the curves of FELM are all at the bottom, and the fluctuations are gentler, while other algorithms have large fluctuations, indicating that compared with other algorithms, it not only obtains the highest accuracy, but also the network outputs of each independent trial are very close to the expected value, and the error is very small.

**TABLE 5:** Comparison of training and testing RMSE.

Data sets	ELM		OP-ELM			OS-RELM		FELM			
								**φ_*1*_**		**φ_*2*_**	
	**Train**	**Test**	**Train**	**Test**	**Final^a^**	**Train**	**Test**	**Train**	**Test**	**Train**	**Test**
Abalone	0.1650	0.1657	0.1486	0.2097	35	0.1850	0.1878	0.0038	**0.0129**	0.0079	**0.0119**
Mpg	0.1825	0.1895	0.1207	0.1605	36	0.2492	0.2608	0.0252	**0.0309**	0.0236	**0.0309**
Autoprice	0.1545	0.1867	0.1272	0.1737	14	0.2113	0.2702	0.0016	**0.0145**	0.0017	**0.0124**
Balloon	0.1533	0.1574	0.0141	0.0237	44	0.1917	0.1925	0.0014	**0.0015**	0.0009	**0.0009**
Baskball	0.2529	0.2817	0.2244	0.2769	7	0.2765	0.2923	0.0119	**0.0342**	0.0195	**0.0402**
Cleveland	0.4247	0.4517	0.4043	0.4331	9	0.5269	0.5632	0.0318	**0.0712**	0.0468	**0.0702**
Cloud	0.1364	0.1685	0.0850	0.1673	18	0.2836	0.3245	0.0023	**0.0120**	0.0054	**0.0103**
Concrete CS	0.2748	0.2786	0.0979	0.1201	85	0.3642	0.3664	0.0136	**0.0150**	0.0150	**0.0160**
Diabetes	0.3483	0.3873	0.2661	0.3764	5	0.3500	0.3788	0.0107	**0.0247**	0.0143	**0.0253**
Housing	0.2353	0.2488	0.1219	0.2341	54	0.2971	0.3122	0.0228	**0.0438**	0.0274	**0.0358**
Machine CPU	0.0800	0.1057	0.0320	0.0943	13	0.1494	0.1783	0.0003	**0.0047**	0.0020	**0.0055**
Mg	0.3385	0.3421	0.2454	0.2707	84	0.3888	0.3923	0.0225	**0.0230**	0.0291	**0.0300**
Quake	0.3445	0.3480	0.3407	0.3462	10	0.3610	0.3614	0.0078	**0.0077**	0.0147	**0.0152**
Servo	0.3565	0.3608	0.1029	0.1956	39	0.4163	0.4268	0.0205	**0.0260**	0.0272	**0.0288**
Strike	0.1579	0.1555	0.1437	0.1557	10	0.2216	0.2180	0.0118	**0.0202**	0.0207	**0.0261**
Wisconsin B.C.	0.0634	0.0920	0.0109	0.0262	48	0.1818	0.2673	0.0007	**0.0279**	0.0023	**0.0225**

^*a*^The number of neurons in the final model.

**TABLE 6:** Comparison of training and testing RMSE.

Data sets	IFELM		SVR		LSSVR		FELM			
							**φ_*1*_**		**φ_*2*_**	
	**Train**	**Test**	**Train**	**Test**	**Train**	**Test**	**Train**	**Test**	**Train**	**Test**
Abalone	0.1882	0.1876	0.1438	0.1658	0.0001	0.1951	0.0038	**0.0129**	0.0079	**0.0119**
Mpg	0.2519	0.2595	0.1031	0.1513	0.0001	0.3549	0.0252	**0.0309**	0.0236	**0.0309**
Autoprice	0.2238	0.2673	0.0332	0.2408	0.0078	0.3737	0.0016	**0.0145**	0.0017	**0.0124**
Balloon	0.1935	0.1906	0.0105	0.0109	0.0003	0.1016	0.0014	**0.0015**	0.0009	**0.0009**
Baskball	0.2705	0.3007	0.1538	0.5060	0.0001	0.3340	0.0119	**0.0342**	0.0195	**0.0402**
Cleveland	0.5353	0.5808	0.0009	0.8334	0.0001	0.5918	0.0318	**0.0712**	0.0468	**0.0702**
Cloud	0.2847	0.3336	0.0233	0.1947	0.0001	0.3476	0.0023	**0.0120**	0.0054	**0.0103**
Concrete CS	0.3679	0.3747	0.0555	0.1008	0.0063	0.4312	0.0136	**0.0150**	0.0150	**0.0160**
Diabetes	0.3584	0.3755	0.1874	0.8771	0.0001	0.3915	0.0107	**0.0247**	0.0143	**0.0253**
Housing	0.3006	0.3226	0.0500	0.2147	0.0001	0.3562	0.0228	**0.0438**	0.0274	**0.0358**
Machine CPU	0.1478	0.1803	0.0026	0.0263	0.0001	0.2138	0.0003	**0.0047**	0.0020	**0.0055**
Mg	0.3777	0.3779	0.2377	0.2794	0.0002	0.4418	0.0225	**0.0230**	0.0291	**0.0300**
Quake	0.3646	0.3660	0.3405	0.3517	0.0955	0.8925	0.0078	**0.0077**	0.0147	**0.0152**
Servo	0.4106	0.4179	0.1552	0.1950	0.0001	0.2187	0.0205	**0.0260**	0.0272	**0.0288**
Strike	0.2207	0.2240	0.1125	0.2053	< 10^−4^	0.1646	0.0118	**0.0202**	0.0207	**0.0261**
Wisconsin B.C.	0.1844	0.2666	0.0003	0.0360	0.0001	0.2100	0.0007	**0.0279**	0.0023	**0.0225**

“IFELM” for “inverse-free ELM”.

**FIGURE 14:**
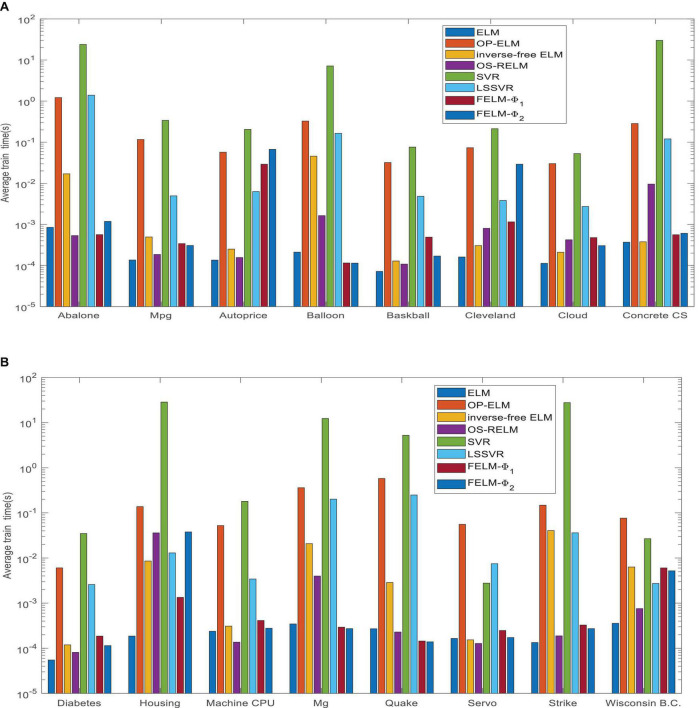
Comparison of eight algorithms training time. **(A)** Eight data sets average training time and **(B)** eight data sets average test time.

Figure 14 shows the average time comparison of 7 algorithms on 16 datasets, where *FELM*−φ_1_ and *FELM*−φ_2_ use φ_*1*_ and φ_*2*_ base functions for FELM, respectively. Tables 7, 8 and Figure 14 show that the average training time of FELM on the two different types of base functions is close, and it is also similar to ELM, inverse-free ELM and OS-RELM in learning speed and test time. But it is obvious from Figure 14 that FELM learns ten times or even more than a hundred times faster than OP-ELM, SVR and LSSVR on most problems.

**TABLE 7:** Comparison of training and testing time.

Data sets	ELM		OP-ELM		OS-RELM		FELM			
							**φ_*1*_**		**φ_*2*_**	
	**Train**	**Test**	**Train**	**Test**	**Train**	**Test**	**Train**	**Test**	**Train**	**Test**
Abalone	0.0008	0.0006	1.2151	0.0053	0.0005	0.0001	0.0006	0.0019	0.0012	0.0058
Mpg	0.0001	0.0003	0.1163	0.0010	0.0002	< 10^−4^	0.0003	0.0004	0.0003	0.0004
Autoprice	0.0001	0.0003	0.0571	0.0004	0.0002	< 10^−4^	0.0291	0.0006	0.0671	0.0006
Balloon	0.0002	0.0003	0.3266	0.0026	0.0016	< 10^−4^	0.0001	0.0003	0.0001	0.0003
Baskball	0.0001	0.0003	0.0319	0.0003	0.0001	< 10^−4^	0.0005	0.0002	0.0002	0.0002
Cleveland	0.0002	0.0003	0.0733	0.0003	0.0008	0.0003	0.0012	0.0007	0.0291	0.0006
Cloud	0.0001	0.0003	0.0301	0.0005	0.0004	< 10^−4^	0.0005	0.0004	0.0003	0.0002
Concrete CS	0.0004	0.0003	0.2841	0.0040	0.0096	0.0001	0.0006	0.0009	0.0006	0.0006
Diabetes	0.0001	0.0003	0.0060	0.0002	0.0001	< 10^−4^	0.0002	0.0001	0.0001	0.0001
Housing	0.0002	0.0003	0.1372	0.0017	0.0360	0.0001	0.0013	0.0008	0.0378	0.0008
Machine CPU	0.0002	0.0003	0.0523	0.0004	0.0001	< 10^−4^	0.0004	0.0003	0.0003	0.0002
Mg	0.0003	0.0003	0.3608	0.0041	0.0040	0.0001	0.0003	0.0006	0.0003	0.0005
Quake	0.0003	0.0003	0.5760	0.0007	0.0002	< 10^−4^	0.0001	0.0004	0.0001	0.0004
Servo	0.0002	0.0002	0.0556	0.0009	0.0001	< 10^−4^	0.0002	0.0002	0.0002	0.0002
Strike	0.0001	0.0002	0.1478	0.0004	0.0002	< 10^−4^	0.0003	0.0005	0.0003	0.0005
Wisconsin B.C.	0.0004	0.0003	0.0763	0.0013	0.0008	0.0001	0.0060	0.0014	0.0052	0.0011

**TABLE 8:** Comparison of training and testing time.

Data sets	IFELM		SVR		LSSVR		FELM			
							**φ_*1*_**		**φ_*2*_**	
	**Train**	**Test**	**Train**	**Test**	**Train**	**Test**	**Train**	**Test**	**Train**	**Test**
Abalone	0.0170	0.0003	23.9456	0.1626	1.3931	0.0829	0.0006	0.0019	0.0012	0.0058
Mpg	0.0005	0.0001	0.3389	0.0018	0.0049	0.0017	0.0003	0.0004	0.0003	0.0004
Autoprice	0.0003	< 10^−4^	0.2050	0.0005	0.0063	0.0046	0.0291	0.0006	0.0671	0.0006
Balloon	0.0458	< 10^−4^	7.1634	0.0282	0.1645	0.0264	0.0001	0.0003	0.0001	0.0003
Baskball	0.0001	< 10^−4^	0.0762	0.0002	0.0048	0.0022	0.0005	0.0002	0.0002	0.0002
Cleveland	0.0003	< 10^−4^	0.2124	0.0014	0.0038	0.0013	0.0012	0.0007	0.0291	0.0006
Cloud	0.0002	< 10^−4^	0.0529	0.0002	0.0027	0.0012	0.0005	0.0004	0.0003	0.0002
Concrete CS	0.0004	0.0002	29.9708	0.0130	0.1204	0.0124	0.0006	0.0009	0.0006	0.0006
Diabetes	0.0001	< 10^−4^	0.0348	0.0001	0.0026	0.0011	0.0002	0.0001	0.0001	0.0001
Housing	0.0086	0.0001	28.4575	0.0044	0.0130	0.0053	0.0013	0.0008	0.0378	0.0008
Machine CPU	0.0003	< 10^−4^	0.1797	0.0006	0.0034	0.0012	0.0004	0.0003	0.0003	0.0002
Mg	0.0208	0.0001	12.3212	0.0160	0.2015	0.0097	0.0003	0.0006	0.0003	0.0005
Quake	0.0029	0.0001	5.2265	0.0331	0.2491	0.0269	0.0001	0.0004	0.0001	0.0004
Servo	0.0002	< 10^−4^	0.0028	0.0002	0.0075	0.0020	0.0002	0.0002	0.0002	0.0002
Strike	0.0405	0.0001	27.6507	0.0050	0.0361	0.0067	0.0003	0.0005	0.0003	0.0005
Wisconsin B.C.	0.0063	0.0009	0.0267	0.0011	0.0027	0.0011	0.0060	0.0014	0.0052	0.0011

“IFELM” for “inverse-free ELM”.

In fact, according to the above experiments, it is obvious that FELM has better generalization performance than other comparison algorithms; at the same time, the RMSE of FELM changes milder or less, which means that FELM has stronger robustness. In addition, ELM has the advantage of fast learning speed, and the algorithm proposed in this article has been shown to not only generalize well, but also compete with ELM in learning speed.

## 4. Conclusions and future works

This article proposes a new type of functional extreme learning machine theory, the parameter learning algorithm without iteration makes the learning speed of FELM very fast. In our simulations, for many problems, the learning stage of FELM can be completed in less than a few seconds. Although the purpose of this article is not to compare functional extreme learning machine with ELM, SVR and their improved algorithms, we also make a simple comparison between FELM and six algorithms in the simulations. The results show that the learning speed of FELM can not only compete with ELM and its improved algorithms, but also be dozens or hundreds of times faster than SVR. As our experimental results show, FELM has higher test accuracy under the same network complexity as ELM and its variants. Because SVR usually generates more support vectors (computing units), LSSVR uses all training data, and functional extreme learning machine just needs few hidden layer nodes (computing units) in the same application. In applications requiring fast prediction and response capability, SVR algorithm may take several hours, so it is not suitable for real-time prediction, and the performance of FELM in this article seems to prove that it is suitable for this application. Compared with popular learning technologies, the proposed FELM has several important characteristics. (1) The training speed of FELM is very fast; (2) Fast parameter learning algorithm without iteration and with high precision; (3) Different function families can be selected according to specific problems, such as trigonometric function bases, Fourier basis functions, etc. In this article, we have proved that FELM is very useful in many practical regression problems, but the following two aspects can be studied in the future: under the actual engineering error, the purpose of optimizing the network is achieved by reducing the network complexity. The network parameters are obtained by matrix pseudo inverse method.

## Data availability statement

The original contributions presented in the study are included in the article/supplementary material, further inquiries can be directed to the corresponding authors.

## Author contributions

XL carried out the FELM algorithm studies, participated in the drafted manuscript. GZ carried out the review and editing. YZ carried out the review and editing. QL carried out the design algorithm model. All authors read and approved the final manuscript.

## References

[B1] Abiodun O. I. Jantan A. Omolara A. E. Dada K. V. Mohamed N. A. Arshad H. (2018). State-of-the-art in artificial neural network applications: A survey. *Heliyon* 4:e00938. 10.1016/j.heliyon.2018.e00938 30519653 PMC6260436

[B2] Afridi M. J. Ross A. Shapiro E. M. (2018). Recent advances in convolutional neural networks. *Pattern Recogn.* 77 354–377. 10.1016/j.patcog.2017.10.013 PMC6377173 30774153

[B3] Artem B. Stefan L. (2017). Extreme learning machines for credit scoring: An empirical evaluation. *Exp. Syst. Appl.* 86 42–53. 10.1016/j.eswa.2017.05.050

[B4] Asuncion A. Newman D. J. (2007). *U machine learning repository, School of Information and Computer Science.* Irvine, CA: University of California.

[B5] Atiquzzaman M. Kandasamy J. (2018). Robustness of Extreme Learning Machine in the prediction of hydrological flow series. *Comput. Geosci.* 120 105–114. 10.1016/j.cageo.2018.08.003

[B6] Baldominos A. Saez Y. Isasi P. (2018). Evolutionary convolutional neural networks: An application to handwriting recognition. *Neurocomputing* 283 38–52. 10.1016/j.neucom.2017.12.049

[B7] Castillo E. (1998). Functional networks. *Neural Process. Lett.* 7 151–159. 10.1023/A:1009656525752

[B8] Christou V. Tsipouras M. G. Giannakeas N. Tzallas A. T. (2018). Hybrid extreme learning machine approach for homogeneous neural networks. *Neurocomputing* 311 397–412. 10.1016/j.neucom.2018.05.064

[B9] Cortes C. Vapnik V. (1995). Support-vector networks. *Mach. Learn.* 20 273–297. 10.1007/BF00994018

[B10] Gautam R. Sharma M. (2019). Speech recognition using deep neural networks: A systematic review. *IEEE Access* 7 19143–19165. 10.1109/ACCESS.2019.2896880 31902041

[B11] Geng Z. Q. Dong J. G. Chen J. Han Y. (2017). A new self-organizing extreme learning machine soft sensor model and its applications in complicated chemical processes. *Eng. Appl. Artif. Intell.* 62 38–50. 10.1016/j.engappai.2017.03.011

[B12] Golestaneh P. Zekri M. Sheikholeslam F. (2018). Fuzzy wavelet extreme learning machine. *Fuzzy Sets Syst.* 342 90–108. 10.1016/j.fss.2017.12.006

[B13] Gong D. Hao W. Gao L. Feng Y. Cui N. (2021). Extreme learning machine for reference crop evapotranspiration estimation: Model optimization and spatiotemporal assessment across different climates in China. *Comput. Electron. Agric.* 187:106294. 10.1016/j.compag.2021.106294

[B14] Guo Z. Yongquan Z. Huajuan H. Zhonghua T. (2019). Functional networks and applications: A survey. *Neurocomputing* 335 384–399. 10.1016/j.neucom.2018.04.085

[B15] Henríquez P. A. Ruz G. A. (2019). Noise reduction for near-infrared spectroscopy data using extreme learning machines. *Eng. Appl. Artif. Intell.* 79 13–22. 10.1016/j.engappai.2018.12.005 31844597

[B16] Huang G. Zhou H. Ding X. Zhang R. (2012). Extreme learning machine for regression and multiclass classification. *IEEE Trans. Syst.* 42 513–529. 10.1109/TSMCB.2011.2168604 21984515

[B17] Huang G. B. Zhu Q. Y. Siew C. K. (2006). Extreme learning machine: Theory and applications. *Neurocomputing* 70 489–501. 10.1016/j.neucom.2005.12.126

[B18] Huang Y. Zhao L. (2018). Review on landslide susceptibility mapping using support vector machines. *Catena* 165 520–529. 10.1016/j.catena.2018.03.003

[B19] Kardani N. Bardhan A. Samui P. Nazem M. Zhou A. Armaghani D. J. (2021). A novel technique based on the improved firefly algorithm coupled with extreme learning machine (ELM-IFF) for predicting the thermal conductivity of soil. *Eng. Comput.* 38 3321–3340. 10.1007/s00366-021-01329-3

[B20] Kärkkäinen T. (2019). Extreme minimal learning machine: Ridge regression with distance-based basis. *Neurocomputing* 342 33–48. 10.1016/j.neucom.2018.12.078

[B21] Li H. T. Chou C. Y. Chen Y. T. Wang S. Wu A. (2019). Robust and lightweight ensemble extreme learning machine engine based on eigenspace domain for compressed learning. *IEEE Trans. Circ. Syst.* 66 4699–4712. 10.1109/TCSI.2019.2940642

[B22] Li S. You Z. Guo H. Luo X. Zhao Z. (2016). Inverse-free extreme learning machine with optimal information updating. *IEEE Trans. Cybern.* 46 1229–1241. 10.1109/TCYB.2015.2434841 26054082

[B23] Lima A. R. Hsieh W. W. Cannon A. J. (2017). Variable complexity online sequential extreme learning machine, with applications to streamflow prediction. *J. Hydrol.* 555 983–994. 10.1016/j.jhydrol.2017.10.037

[B24] Miche Y. Sorjamaa A. Bas P. Simula O. Jutten C. Lendasse A. (2010). OP-ELM: Optimally pruned extreme learning machine. *IEEE Trans. Neural Netw.* 21 158–162. 10.1109/TNN.2009.2036259 20007026

[B25] Mohammed E. Hossam F. Nadim O. (2018). Improving Extreme Learning Machine by Competitive Swarm Optimization and its application for medical diagnosis problems. *Exp. Syst. Appl.* 104 134–152. 10.1016/j.eswa.2018.03.024

[B26] Murli M. Ebha K. Subhojit G. (2018). Microgrid protection under wind speed intermittency using extreme learning machine. *Comput. Electr. Eng.* 72 369–382. 10.1016/j.compeleceng.2018.10.005

[B27] Ozgur K. Meysam A. (2018). Modelling reference evapotranspiration using a new wavelet conjunction heuristic method: Wavelet extreme learning machine vs wavelet neural networks. *Agric. For. Meteorol.* 263 41–48. 10.1016/j.agrformet.2018.08.007

[B28] Pacheco A. G. C. Krohling R. A. Da Silva C. A. S. (2018). Restricted Boltzmann machine to determine the input weights for extreme learning machines. *Exp. Syst. Appl.* 96 77–85. 10.1016/j.eswa.2017.11.054 31880561

[B29] Paolo P. Roberto F. (2017). Application of extreme learning machines to inverse neutron kinetics. *Ann. Nuclear Energy* 100 1–8. 10.1016/j.anucene.2016.08.031

[B30] Peter S. Israel C. (2020). Extreme learning machine for a new hybrid morphological/linear perceptron. *Neural Netw.* 123 288–298. 10.1016/j.neunet.2019.12.003 31891839

[B31] Sattar A. Ertuğrul ÖF. Gharabaghi B. McBean E. A. Cao J. (2019). Extreme learning machine model for water network management. *Neural Comput. Appl.* 31 157–169. 10.1007/s00521-017-2987-7

[B32] Shao Z. Er M. J. (2016). An online sequential learning algorithm for regularized extreme learning machine. *Neurocomputing* 173 778–788. 10.1016/j.neucom.2015.08.029

[B33] StatLib DataSets Archive (2021). *StatLib DataSets Archive.* Available online at: https://www.causeweb.org/cause/resources/library/r12673

[B34] Sun H. Jia J. Goparaju B. Huang G. Sourina O. Bianchi M. (2017). Large-scale automated sleep staging. *Sleep* 40:zsx139. 10.1093/sleep/zsx139 29029305 PMC6251659

[B35] Tang J. Deng C. Huang G. B. (2016). Extreme learning machine for multilayer perceptron. *IEEE Trans. Neural Netw. Learn. Syst.* 27 809–821. 10.1109/TNNLS.2015.2424995 25966483

[B36] Vikas D. Balaji S. (2020). Physics informed extreme learning machine (PIELM)–A rapid method for the numerical solution of partial differential equations. *Neurocomputing* 391 96–118. 10.1016/j.neucom.2019.12.099

[B37] Werbos P. (1974). *New tools for prediction and analysis in the behavioral sciences.* Ph. D. thesis. Cambridge, MA: Harvard University.

[B38] Yaseen Z. M. Sulaiman S. O. Deo R. C. Chau W. K. (2019). An enhanced extreme learning machine model for river flow forecasting: State-of-the-art, practical applications in water resource engineering area and future research direction. *J. Hydrol.* 569 387–408. 10.1016/j.jhydrol.2018.11.069

[B39] Yimin Yang Q. M. Jonathan W. (2018). Autoencoder with invertible functions for dimension reduction and image reconstruction. *IEEE Trans. Syst. Man Cybern.* 48 1065–1079. 10.1109/TSMC.2016.2637279

[B40] Zhang Y. Kuang Z. Xiao X. (2009a). A direct-weight-determination method for trigonometrically- activated fourier neural networks. *Comput. Eng. Sci.* 31 112–115.

[B41] Zhang Y. Liu W. Cai B. (2009b). Number determination of hidden-layer neurons in weights-directly-determined Legendre neural network. *J. Chin. Comp. Syst.* 30 1298–1301.

[B42] Zong W. W. Huang G. B. Chen Y. Q. (2013). Weighted extreme learning machine for imbalance learning. *Neurocomputing* 101 229–242.

